# Developments in Behavioral Neuroscience as Reflected by the Most Viewed and Highly Cited Articles Published in Brain Sciences in 2025

**DOI:** 10.3390/brainsci16090917

**Published:** 2026-08-28

**Authors:** Luigi De Gennaro

**Affiliations:** Department of Psychology, University of Rome Sapienza, 00185 Roma, Italy; luigi.degennaro@uniroma1.it

## 1. Introduction

Each year, *Brain Sciences* identifies the most downloaded, viewed, and cited articles for each section and invites the Section Editor to compile a selection based on originality, methodological rigor, relevance to current research trends, and overall scholarly impact.

Below are our top six choices. It is worth highlighting certain unexpected trends. While the Section’s scope predictably emphasizes the understanding of neural mechanisms underlying behavior, the top two positions in our 2025 selection are occupied by a narrative and a systematic review that are not directly and immediately related to such scope. In our view, this is not an anomaly but rather signals the need to become used to the fact that in peer-reviewed scientific publishing, it is also appropriate to consider something we can call “social engagement”. The two reviews highlighted here fall into this category, all while maintaining the rigorous standards of classic neuroscientific reviews.

It indicates a way of conducting scientific research that is deeply embedded in the broader world, with an effort to shorten the distance between field experts and society at large.

In the list of choices, there are also more “conventional” articles, pointing out once again a tendency to consider reviews among the most relevant articles. On the one hand, this is almost expected in terms of citations, downloads, or views. On the other hand, it indicates the need to identify new, supplementary criteria to evaluate the quality of a single article that do not reflect this intrinsic bias.

## 2. Heterosexual Intimate Partner Femicide: A Narrative Review of Victim and Perpetrator Characteristics

This narrative review by Koureta et al. focuses on interpersonal partner femicide (IPF), the most severe manifestation of gender-based violence and a critical public health issue rooted in global gender inequality [1]. A narrative synthesis of existing research reveals that this act is not unpredictable, as multiple identifiable risk indicators consistently emerge. Victims frequently have histories of prior psychological and physical abuse, while perpetrators tend to exhibit patterns of psychiatric disorders, substance use, jealousy, stalking, and rigid patriarchal beliefs, often triggered by separation or ongoing conflict. Their profile differs from other homicide offenders in distinct ways, yet heterogeneity among them remains. The review concludes that timely risk assessment, early intervention in cases of intimate partner violence, and coordinated responses among law enforcement, social services, and mental health professionals are essential for identifying at-risk women and ultimately preventing future femicides.

## 3. AI Chatbots and Cognitive Control: Enhancing Executive Functions Through Chatbot Interactions: A Systematic Review

In this systematic review, Pergantis et al. explore how conversational agents (AI chatbots) might reinforce executive functions—the higher-order mental skills required for planning, strategizing, and achieving goals. AI chatbots show genuine promise for strengthening functions like planning and goal setting, with research pointing to positive effects on cognitive and social development [2]. However, the field remains in its early stages, as current studies face significant limitations that make their findings difficult to generalize or replicate. The application of chatbots for executive skills training remains preliminary, and the authors highlight the need for a unified framework, more robust study designs, diverse populations, larger sample sizes, and longitudinal studies to observe long-term effects.

## 4. The Impact of Immediate and Delayed Rewards on Task-Switching Performance

This original study by Zhao et al. investigated how immediate versus delayed rewards differentially affect cognitive performance during task repetition and task switching, while also exploring the underlying neural mechanisms of attention allocation using event-related potentials. Behavioral and electrophysiological (P300) findings converged to show that immediate rewards significantly enhanced performance and elicited larger neural responses compared to delayed rewards, but notably, this advantage occurred exclusively during repeated trials; under task-switching conditions, no differences between reward types were observed. The results suggest that rewards facilitate task performance by optimizing attentional focus on the ongoing activity. However, when task-switching demands require substantial cognitive resources to process multiple cues simultaneously, the remaining capacity becomes insufficient to effectively process reward-related information, thereby canceling out the reward benefit. According to the Expected Value of Control theory [3], this study suggests that the efficacy of reward depends on the dynamic allocation of limited attentional resources across varying cognitive demands. The key implication is that, when multiple cues are processed simultaneously, participants must adjust their cognitive strategies to optimize performance and secure greater rewards.

## 5. The Brain That Understands Diversity: A Pilot Study Focusing on the Triple Network

This pilot study investigated whether an individual’s gray matter volume—considered an indicator of brain health—relates to their understanding and acceptance of diversity in terms of gender, sexuality, and origin. Gray matter volume was assessed through MRI-based analysis, while participants’ attitudes toward diversity were measured using psychological questions drawn from the World Values Survey. The authors focused on the triple network model [4], encompassing the saliency network (SN), the central executive network (CEN), and the default mode network (DMN), which is critical to empathic processing. The findings revealed that individuals with the highest level of diversity understanding exhibited greater gray matter volume at the whole-brain level, particularly within networks associated with rational judgment, emotion regulation, and self- and other-awareness, with similar trends observed in the network linked to introspection. In contrast, the remaining participants showed no such deviations from expected volume. These differences persisted even when accounting for educational and occupational backgrounds. The authors conclude that a healthy brain, especially involving core networks that govern cognitive control, emotional processing, and social awareness, may be foundational to embracing human diversity. Of great importance, this pilot study provides the first evidence of a structural neural basis underlying the acceptance of diverse people.

## 6. Cognitive Behavioral Therapy (CBT) for Managing Tinnitus, Hyperacusis, and Misophonia: The 2025 Tonndorf Lecture

This perspective paper summarizes key points from the 2025 Tonndorf Lecture presented by Hashir Aazh at the third World Tinnitus Congress and the 14th International Tinnitus Seminar in Poland [5]. Cognitive Behavioral Therapy (CBT) is a validated intervention for distress caused by tinnitus, hyperacusis, and misophonia, with proven efficacy whether delivered by psychologists, audiologists, or through guided digital platforms (unguided versions also work but suffer from higher dropout). Despite its effectiveness, the response is not universal—some patients remain distressed post-treatment—yet the actual need for intensive therapy is modest, estimated at roughly 1 in 52 tinnitus sufferers [6]. To advance care, future research should directly compare provider-led models, test hybrid in-person and digital approaches, and tackle digital CBT’s specific limitations for hyperacusis and misophonia. Incorporating neuroimaging and physiological data into trials could clarify neural mechanisms of improvement, while systematically profiling non-responders would enable more personalized, targeted therapeutic strategies.

## 7. Eye-Tracking Metrics as a Digital Biomarker for Neurocognitive Disorders in Multiple Sclerosis: A Scoping Review

Although eye-tracking has increasingly been used to study cognitive decline in multiple sclerosis (MS) [7], the wide heterogeneity in paradigms and methodologies across studies has hindered a clear, comprehensive understanding of its clinical utility. To address this gap, this scoping review by Cecchetti et al. maps the existing literature to identify which oculomotor metrics are most relevant to the neurocognitive profiles of people with MS. Results indicate that anti-saccade latency and error rates are the most frequently proposed metrics, showing significant correlations with standard cognitive measures and strongly suggesting that eye tracking holds promise for early detection of neurocognitive disorders in clinical practice. Nevertheless, the findings underscore an urgent need for primary, multidisciplinary research that fully captures the complexity of MS across its various phenotypes and disease-related variables. Regarding the causal relationship, future investigations must clarify whether oculomotor dysfunction in MS serves as a precursor to cognitive deficits or merely follows their onset.

These selected articles present a highly varied range of topics and, as anticipated, are somewhat unusual for the specific field of Behavioral Neuroscience. They share the common goal of identifying promising and often original research areas also maintaining a focus on public engagement.

At this point, all that remains for us is to encourage readers to explore these valuable contributions and contribute to further research in this field.
